# Clinical and genetic associations of asymmetric apical and septal left ventricular hypertrophy

**DOI:** 10.1093/ehjdh/ztae060

**Published:** 2024-08-09

**Authors:** Victoria Yuan, Milos Vukadinovic, Alan C Kwan, Florian Rader, Debiao Li, David Ouyang

**Affiliations:** School of Medicine, University of California, Los Angeles, CA, USA; Department of Cardiology, Smidt Heart Institute, Cedars-Sinai Medical Center, 127 S San Vicente Blvd, Los Angeles, CA 90034, USA; Samueli Bioengineering, University of California, Los Angeles, CA, USA; Department of Cardiology, Smidt Heart Institute, Cedars-Sinai Medical Center, 127 S San Vicente Blvd, Los Angeles, CA 90034, USA; Department of Cardiology, Smidt Heart Institute, Cedars-Sinai Medical Center, 127 S San Vicente Blvd, Los Angeles, CA 90034, USA; Cedars-Sinai Medical Center, Biomedical Imaging Research Institute, Los Angeles, CA, USA; Department of Cardiology, Smidt Heart Institute, Cedars-Sinai Medical Center, 127 S San Vicente Blvd, Los Angeles, CA 90034, USA; Division of Artificial Intelligence in Medicine, Department of Medicine, Cedars-Sinai Medical Center, 127 S San Vicente Blvd, Los Angeles, CA 90034, USA

**Keywords:** Left ventricular hypertrophy, Hypertrophic cardiomyopathy, Apical hypertrophy, Septal hypertrophy

## Abstract

**Aims:**

Increased left ventricular mass has been associated with adverse cardiovascular outcomes including incident cardiomyopathy and atrial fibrillation. Such associations have been studied in relation to total left ventricular hypertrophy, while the regional distribution of myocardial hypertrophy is extremely variable. The clinically significant and genetic associations of such variability require further study.

**Methods and results:**

Here, we use deep learning–derived phenotypes of disproportionate patterns of hypertrophy, namely, apical and septal hypertrophy, to study genome-wide and clinical associations in addition to and independent from total left ventricular mass within 35 268 UK Biobank participants. Using polygenic risk score and Cox regression, we quantified the relationship between incident cardiovascular outcomes and genetically determined phenotypes in the UK Biobank. Adjusting for total left ventricular mass, apical hypertrophy is associated with elevated risk for cardiomyopathy and atrial fibrillation. Cardiomyopathy risk was increased for subjects with increased apical or septal mass, even in the absence of global hypertrophy. We identified 17 genome-wide associations for left ventricular mass, 3 unique associations with increased apical mass, and 3 additional unique associations with increased septal mass. An elevated polygenic risk score for apical mass corresponded with an increased risk of cardiomyopathy and implantable cardioverter-defibrillator implantation.

**Conclusion:**

Apical and septal mass may be driven by genes distinct from total left ventricular mass, suggesting unique genetic profiles for patterns of hypertrophy. Focal hypertrophy confers independent and additive risk to incident cardiovascular disease. Our findings emphasize the significance of characterizing distinct subtypes of left ventricular hypertrophy. Further studies are needed in multi-ethnic cohorts.

## Graphical Abstract

**Graphical Abstract:**
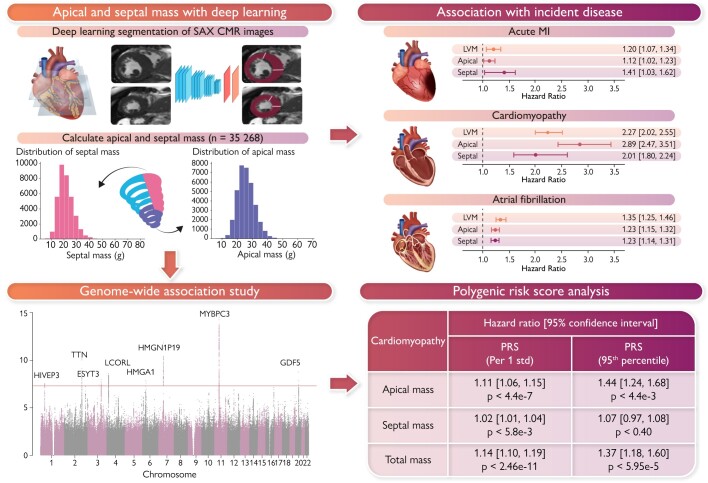

## Introduction

Left ventricular (LV) mass (LVM) is associated with genetic variants of hypertrophic cardiomyopathy (HCM) and dilated cardiomyopathy (DCM) and cardiovascular outcomes such as stroke, arrhythmias, and sudden cardiac death.^1,2^ Increased total LVM can reflect disease progression, such as long-standing hypertension or valvular disease, while disproportionate or early-onset hypertrophy can provide additional insight into genetic aetiologies of myocardial hypertrophy. While there are known clinical patterns of asymmetric hypertrophy,^3–5^ including septal and apical hypertrophy patterns^6–8^ in HCM,^1,6,7^ there are few studies detailing the genetic determinants of focal or disproportionate hypertrophy.^8^ Yet patients with apical HCM face higher rates of atrial fibrillation and apical aneurysms and have different risk factors for sudden cardiac death compared with those with asymmetric septal HCM.^3,7^ Moreover, patients with apical HCM are less likely to report a positive family history, suggesting differences in pathophysiology. Studies of asymmetric cardiomyopathy have been limited by cohort size and incomplete characterization by echocardiography, such that the genetics of patterns of increased mass are not thoroughly explored.^6,8,9^

Deep learning–enabled high-throughput evaluation of cardiac imaging opens an avenue for large-scale studies of cardiac phenotypes.^10–12^ The ability to precisely phenotype cardiac imaging^13^ and combine underlying genetic information allows for the interrogation of a wide range of phenotypes and clinically meaningful traits. The UK Biobank (UKBB) initiative, particularly with the imaging cohorts which include cardiac magnetic resonance (CMR) imaging, has been leveraged to understand the genetics of cardiovascular form and function.^10,14,15^ With high fidelity imaging with CMR, deep learning can precisely and reproducibly evaluate imaging structures.^5,11,13^ Previous studies have already evaluated the genetics of LV wall thickness and mass,^10,14,15^ finding strong associations with *TTN* and *CDKN1A* among other variants. However, these efforts analyse the LV as a whole, rather than evaluating focal patterns of the LV, such as disproportionate apical or septal mass. While variations in apical wall thickness of UKBB participants have been quantified, its genetic and clinical implications have not been characterized.^5^ In this work, we sought to build upon this foundation to evaluate the genetic underpinnings and clinical impact of focal hypertrophy.

Distinguishing between subtypes of LV hypertrophy (LVH) may improve risk stratification^2,7^ and motivate targeted therapies.^16^ Our study leveraged a deep learning algorithm to segment short-axis CMR images^13^ and estimate total LVM, apical LVM, and septal mass for 35 268 UKBB participants. The algorithm has accuracy comparable with human performance, with a Dice metric of 0.88 for identifying the LV myocardium.^13^ These quantifications were used to examine the genetic basis for asymmetric LVM, develop polygenic risk scores (PRSs), and analyse the associations and additional risk of isolated septal and apical hypertrophy with cardiovascular outcomes. Expression quantitative trait loci (eQTLs), gene set analysis, and chromatin interaction mapping were performed to further characterize the functional impact of these loci.

## Methods

### UK Biobank

The UKBB is a prospective study with 502 461 participants from 40 to 60 years of age. Detailed non-imaging data, such as genotyping, diagnoses, and environmental factors, are also included. Our analysis focused on the 45 361 individuals who underwent CMR imaging.

### Measurement of apical and interventricular septal mass

We leveraged a fully convolutional neural network to segment the end-diastolic short-axis CMR images of 45 361 individuals in the UKBB. Participants were excluded based on image quality and visualization of the ventricles and apex (see Supplementary material online, *Figure S1*). We established our final study cohort (*n* = 35 268) to measure apical and interventricular septal mass. Apical mass was calculated by isolating the pixels corresponding to the LV myocardial wall in the last four slices. The pixels were then summed to obtain apical area and then multiplied by slice thickness and slice gap to calculate apical volume. Finally, the volume was multiplied by myocardial density (1.055 g/cm^3^) to obtain apical mass. For each slice, we defined the septum as the segment of the LV myocardium bounded by the insertion points of the right ventricle wall. The pixels were summed to calculate the septal area and then multiplied by slice thickness, slice gap, and myocardial density to measure septal mass.

### Testing for associations with incident disease

We analysed associations between CMR-derived LVM, apical mass, and septal mass with cardiomyopathy, atrial fibrillation, and acute myocardial infarction (MI). We also examined the relationship between these outcomes and global, apical, and septal hypertrophy. Based on current clinical definitions, we defined global hypertrophy as individuals with overall LVM above the sex-specific 90th percentile.^17^ Similarly, apical hypertrophy was defined as individuals with 90th percentile of apical mass and septal hypertrophy as the 90th percentile of septal mass. Cox proportional hazards analysis was performed adjusted for sex, age pulse rate, and hypertension. Incident disease was identified using the reported International Classification of Disease 9th and 10th codes available in UKBB starting from the time of CMR acquisition until the date of the first incident, death, or the last follow-up. Prevalent cases were identified as subjects whose date of first incident was prior to the date of assessment for participation in UKBB. Prevalent cases were removed.

To analyse apical mass, septal mass, and LVM as continuous variables, we leveraged Cox proportional hazards testing in three different models. The first model investigated each mass phenotype individually as independent risk factors while adjustments for sex, age at magnetic resonance imaging (MRI) acquisition, pulse rate, and hypertension. The second model jointly analysed apical mass and LVM, with the same adjustments as the first model. The third model jointly analysed septal mass and LVM. To test associations between cardiomyopathy and ventricular hypertrophy, we created seven different cohorts.

### Genome-wide association study

Given our cohort of 35 268 individuals, 847 were removed due to incomplete or low-quality genetic data. We then performed genome-wide association studies (GWAS) of total LVM, apical mass, and septal mass on 34 421 individuals with BOLT-LMM v2.3.4,^18^ which uses a Bayesian mixture prior as a random effect to fit a linear mixed model. We utilized the UKBB-imputed genotype calls in BGEN v1.2 format. Variants were required to have a minor allele frequency ≥ 0.01, and imputed variants had an INFO score ≥ 0.3. Our model was adjusted for age at CMR imaging, sex, and the first 10 principal components of genetic ancestry. Variants were considered statistically significant at the standard genome-wide significance level of *P* = 5e-8. Independent significant single nucleotide polymorphisms (SNPs) were then defined as SNPs that met the threshold for genome-wide significance, had an *r*^2^ > 0.6, and were located at least ±500 kb away from each other.

### Functional annotation of significant loci

We utilized FUMA v1.6.0^19^ to investigate eQTL, 3D chromatin interaction mapping, and gene set analysis to better understand the function of genome-wide significant variants. First, using GTEx version 8 eQTL tissue data, we evaluated the relationship between SNPs of genome-wide significance with cardiac gene expression in the aorta, atrial appendage, and LV tissue. GTEx contains pre-calculated false discovery rates (FDR) for gene–tissue pairs. FUMA defines significant eQTLs as SNP–gene pairs with *P* < 0.05 and gene–tissue pairs with an FDR ≤ 0.05. Candidate genes were selected using proximity to lead variants, eQTL analysis, cardiac tissue–specific expression, and existing studies. Colocalization of eQTL and GWAS was performed using transcriptome-wide association studies (TWAS) with S-PrediXcan.^20^ Data were first harmonized, and a prediction model using multivariate adaptive shrinkage was then developed with training sets of GTEx genotypes and normalized expression data in the atrial appendage and LV tissues. Training sets were provided by S-PrediXcan. We performed gene set analysis using 10 678 curated gene sets annotated with Gene Ontology terms; Bonferroni correction was performed for all gene sets. Significant gene sets were those with a *P*_Bon_ < 0.05.

### Polygenic risk score analysis

Using PRScs, which utilizes continuous shrinkage priors to infer SNP effect sizes, a polygenic risk model with 155 094 variants was derived from the GWAS summary statistics for apical mass, septal mass, and total LVM. A PRS was then generated for 424 697 UKBB participants who did not undergo CMR imaging and were thus not included to derive the polygenic risk model. With Cox proportional hazards model, we then evaluated the relationship between genetically predicted apical mass and the following cardiovascular outcomes: atrial fibrillation, acute MI, cardiomyopathy, and implantable cardioverter-defibrillator (ICD) implantation. Models were adjusted for sex, age at acquisition, body mass index, pulse rate, and hypertension. Similarly to previous analyses, subject outcomes were using the obtained with International Classification of Disease 9th and 10th codes available in UKBB starting from the time of CMR acquisition until the date of the first incident, death, or the last follow-up.

## Results

### Characterizing apical and septal mass in the UK Biobank cohort

**Figure 1:**
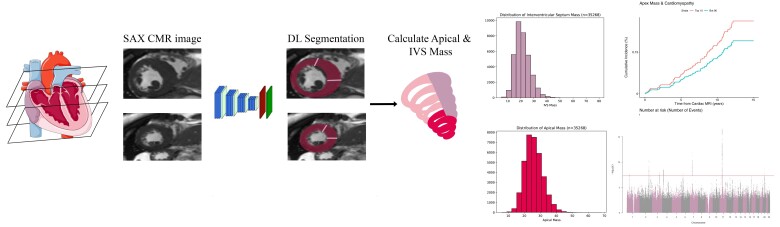
(*A*) Flow diagram to create the cohort for genetic and phenotypic analysis. (*B*) We characterized a cohort of 35 268 participants for total left ventricular, apical, and septal mass using deep learning. From these derived traits, we performed genome-wide association studies to identify genetic drivers of these phenotypes and analysed the relationship between apical mass, septal mass, and incident cardiovascular disease.

**Figure 2:**
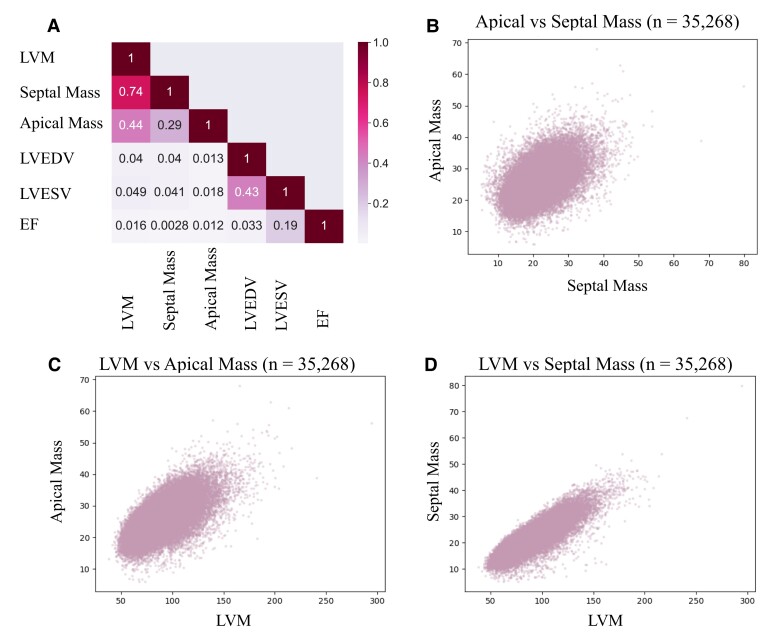
(*A*) Heatmap showing the correlation between apical mass, septal mass, left ventricular mass, left ventricular end systolic volume, left ventricular end diastolic volume, left ventricular ejection fraction. Relationship between (*B*) apical and septal mass, (*C*) apical and LVM, and (*D*) septal mass and left ventricular mass in the cohort.

We established a cohort of 35 268 individuals with measurable total LV, apex, and septum mass from the short-axis CMR images (*Figure 1*). Subjects were excluded if images were low quality or did not fully demonstrate the apex or septum (*Figure 1C*). Left ventricular septal and apical mass were both normally distributed with higher mean values observed among males (see Supplementary material online, *Figure S1*). There was modest correlation between apical mass and total LVM (*r*^2^ = 0.44) but a higher correlation between septal mass and LVM (*r*^2^ = 0.74). Neither septal mass nor apical mass was significantly correlated with other traditional imaging measurements of the LV (*Figure 2*). There was a low prevalence of pre-existing aortic valve disease, hypertension, and cardiomyopathy in the cohort (see Supplementary material online, *Figure S1*).

### Focal hypertrophy’s association with incident cardiovascular disease

**Figure 3:**
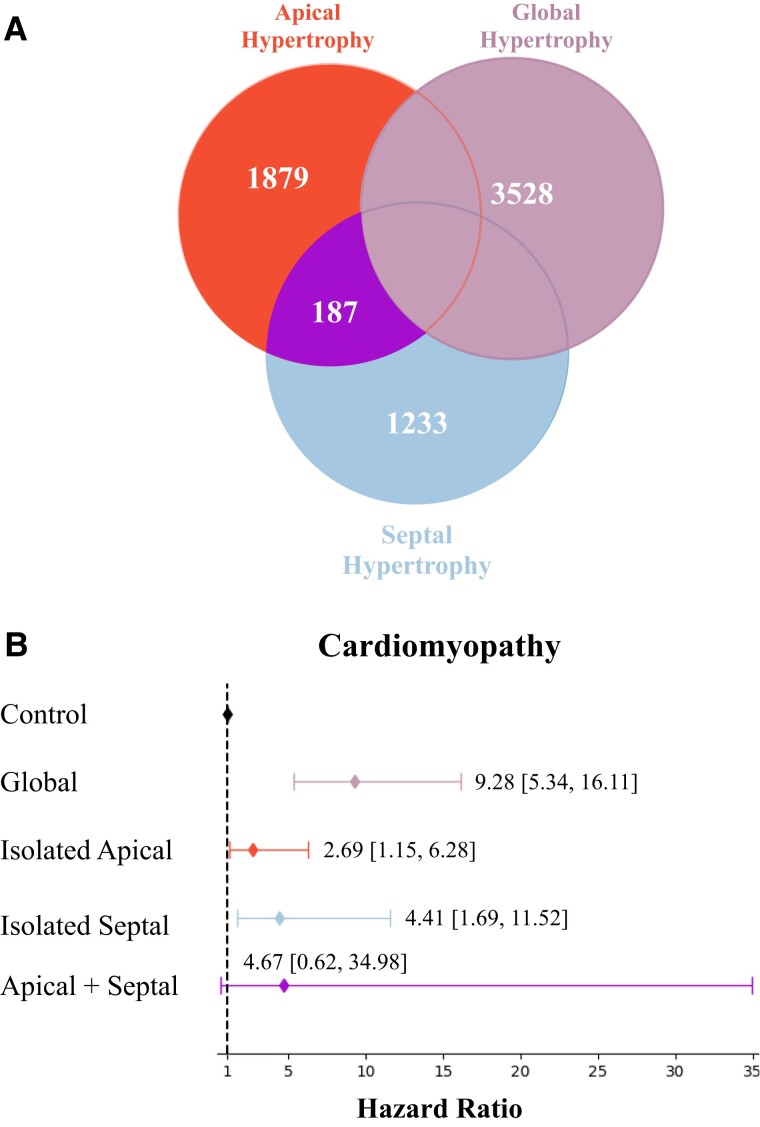
Each category of hypertrophy was defined as sex-specific 90th percentile of mass. (*A*) Venn diagram displaying global hypertrophy, isolated apical hypertrophy, isolated septal hypertrophy, and combined apical and septal hypertrophy. (*B*) Hazard ratios and 95% confident intervals for each phenotype with regard to cardiomyopathy. Subjects without hypertrophy (*n* = 28 441) served as the control group.

Given the correlation between focal regional mass and total LVM, we assessed the individual and combined contribution of increased mass and its association with incident cardiomyopathy (*Figure 3*). We first investigated increased mass categorically by using a binary classification to stratify patients by those with hypertrophy vs. those without. Hypertrophy was defined as ventricular mass exceeding sex-specific 90th percentile.^10,21^ We identified subjects with global LVH (where total LVM was greater than sex-specific 90th percentile), isolated apical hypertrophy without global hypertrophy, and isolated septal hypertrophy without global hypertrophy. Subjects with LVH due to rare variants of HCM and DCM were not excluded, as existing studies with the UKBB cohort have demonstrated that rare variant information is not available for all individuals and many cases of clinically confirmed HCM or DCM often do not have an identified causal variant.^10^ Using a Cox proportional hazards model, subjects with global hypertrophy had the highest risk of cardiomyopathy [hazard ratio (HR) = 9.28, 95% confidence interval = (5.34–16.11), *P* < 2.48e-15]. In parallel, isolated apical hypertrophy without the presence of global hypertrophy also conferred a higher risk of cardiomyopathy [HR = 2.69 (1.15–6.28), *P* < 0.02], and isolated septal hypertrophy conferred a higher risk [HR = 4.41 (1.69–11.53), *P* < 0.002]. Our findings suggest that isolated regional hypertrophy confers excess risk for incident cardiomyopathy independent of global hypertrophy.

In addition to binary classification as hypertrophy, we investigated whether quantitative apical mass and septal mass are independent risk factors for incident cardiovascular disease. Using a Cox proportional hazards model, we found that increased apical, septal, and total mass predicts incident cardiomyopathy, atrial fibrillation, and MI (see Supplementary material online, *Figure S2*). One standard deviation increase in LVM (22.29 g) was associated with a HR of 2.27 (2.02–2.55, *P* < 2.0e-16) for increased risk for cardiomyopathy, and one standard deviation increase in apical mass (5.58 g) was associated with a HR of 2.89 (2.47–3.51, *P* < 2.0e-16) for increased risk for cardiomyopathy. To test whether increased focal mass has independent predictive value beyond total LVM, we performed Cox analysis with models including both LVM and apical mass (M^LVM + apical^) and both LVM and septal mass (M^LVM + septal^). In M^LVM + apical^, in addition to a significant HR for total LVM, apical mass was also an independent predictor of cardiomyopathy and atrial fibrillation. In contrast, in M^LVM + septal^, the HR for septal mass completely attenuated with adjustment for total LVM. Our results suggest that septal mass predicts incident disease by proxying total LVM, while apical mass provides additive independent predictive value for incident cardiovascular disease.

### Genome-wide studies of cardiac magnetic resonance–derived total left ventricular mass, apical mass, and septal mass

**Figure 4:**
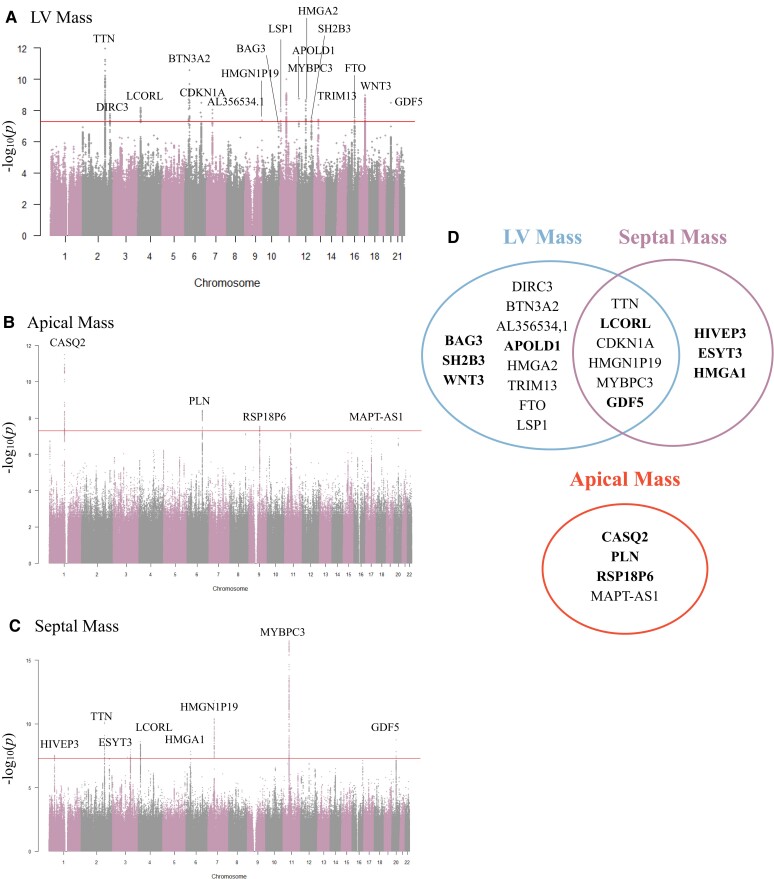
Manhattan plots for genome-wide association studies of (*A*) left ventricular mass, (*B*) apical mass, and (*C*) septal mass. The horizontal red line represents a genome-wide significant *P*-value of 5e-8. (*D*) Significant hits are compared across the three phenotypes, with novel loci in bold.

We performed GWAS of apical and septal mass in 34 421 individuals who met CMR image and genetic quality criteria and compared the results with the genetic associations of total LVM (*Figure 4*), with quantile–quantile plots for each GWAS in Supplementary material online, *Figure S3*. For total LVM, 17 independent variants reached genome-wide significance (*Table 1*), including six loci linked to six genes that were previously recognized to be associated with cardiovascular disease. *BAG3*, *BTN3A2*, and other identified genes include those previously associated with HCM, restrictive cardiomyopathy, mitral valve disease, and LV dilation.^22–31^ Genome-wide association studies of septal mass identified nine loci at genes shared with total LVM, with septal mass and total LVM sharing the same loci for *HMGN1P19.* Three loci for septal mass were associated with three unique genes—*HIVEP3*, *CEP70*, and *HMGA1*—that have not been correlated with LV wall thickness or LVM.^10,15^ For apical mass, we identified three distinct loci—at genes *CASQ2*, *PLN*, and *MAPT*—that were not associated with total LVM or septal mass and that have not been previously associated with apical hypertrophy.^3,32^ Of note, *CASQ2* and *PLN* were not correlated with LV wall thickness^15^ or LVM.^10^

**Table 1:** Genome-wide significant variants and candidate genes across all phenotypes, with novel genes in bold

Phenotype	Chr	Candidate gene	hg37 position	Ref	Alt	*P*-value	Beta
Apex	1	**CASQ2**	116 297 758	A	C	3.2e-12	−0.262
Apex	6	**PLN**	118 876 539	T	C	3.7e-9	−0.220
Apex	9	**RSP19P6**	83 396 102	C	T	2.7e-8	0.396
Apex	17	MAPT	43 948 347	C	T	3.3e-8	−0.224
Septal	1	**HIVEP3**	42 007 188	A	C	2.9e-8	0.192
Septal	2	TTN	179 839 888	T	C	6.2e-11	0.587
Septal	3	**CEP70**	138 172 951	A	G	1.8e-8	−0.193
Septal	4	**LCORL**	18 034 463	G	A	3.2e-9	0.295
Septal	6	**HMGA1**	34 190 104	A	G	2.00e-8	−0.398
Septal	7	**HMGN1P19**	46 620 312	T	C	2.50e-12	−0.242
Septal	11	MYBPC3	47 366 095	T	G	2.20e-14	−0.386
Septal	20	**GDF5**	34 025 756	G	A	1.00e-9	−0.211
Total	2	TTN	179 672 414	T	C	2.1e-11	1.850
Total	2	**DIRC3**	218 288 831	T	A	3.50e-8	−0.674
Total	4	**LCORL**	17 917 781	C	A	1e-8	0.786
Total	6	**BTN3A2**	26 327 814	C	T	2.10e-9	0.729
Total	6	CDKN1A	36 646 849	C	T	4.30e-11	−0.869
Total	6	**AL356534.1**	127 184 986	A	G	3.80e-9	−0.747
Total	7	**HMGN1P19**	46 620 312	T	C	1.10e-8	−0.706
Total	10	**BAG3**	121 415 685	A	G	4.60e-8	0.815
Total	11	**LSP1**	1 902 768	A	G	7.8e-9	−0.729
Total	11	MYBPC3	47 427 180	AGA	A	1.3e-10	−1.166
Total	12	**APOLD1**	12 883 632	A	AG	1.60e-9	0.966
Total	12	HMGA2	66 343 400	C	G	4.00e-9	0.717
Total	12	**SH2B3**	111 884 608	C	G	3.8e-8	−0.669
Total	13	TRIM13	50 565 104	A	ACT	5.7e-9	−2.538
Total	16	FTO	53 812 770	A	ATTTT	2.90e-8	−0.691
Total	17	**WNT3**	44 862 613	G	A	9.30e-10	0.873
Total	20	**GDF5**	34 025 756	G	A	1.50e-9	−0.744

### Functional investigation of markers for apical and septal mass

We leveraged eQTL analysis and gene set analysis to annotate the functions of significant variants of apical and septal mass. Expression quantitative trait locus analysis of apical mass variants reveals that the lead variant of *CASQ2* is associated with increased expression in the left ventricle, atrial appendage, and coronary artery (see Supplementary material online, *Table S4*). The lead variant for *PLN* is associated with increased expression in the aorta and atrial appendage, while the lead variant for *MAPT* is associated with increased expression in the LV. Using TWAS, predicted expression of *CASQ2* was significantly associated with apical mass in the LV (*P* = 1.47e-7), atrial appendage (*P* = 3.30e-10), and coronary artery (*P* = 1.50e-7) and *PLN* in the atrial appendage only (*P* = 3.65e-9). To further investigate the physiological function of genes associated with apical mass, we performed gene set analysis. We found strong associations with networks for cardiomyocyte contraction by calcium ion signalling (*P* = 4.01e-5), regulation of sequestered calcium ion release for cardiac muscle contraction (*P* = 5.25e-5), and cell communication by electrical coupling (*P* = 6.54e-6).

Applying this analysis to variants governing septal mass, we found that *HIVEP3* interacts with *EDN2*, a risk factor for atrial fibrillation in individuals with HCM.^33^*GDF5*, which is shared between the total LV and septal mass phenotypes, has chromatin contacts with *MYH7B*, which in turn activates the CaMK signalling pathway involved in the pathophysiology of HCM.^34^ The lead variant of *CEP70* is associated with enriched expression in the coronary artery and LV (see Supplementary material online, *Table S4*), and TWAS-predicted expression of *CEP70* in the LV is associated with septal mass (*P* = 9.42e-8). Finally, with gene set analysis, we discovered that genes governing septal mass are significantly associated with pathways for the morphogenesis and development of the atria (*P*_Bon_ = 0.0087), septum (*P*_Bon_ = 0.0122), and striated muscle (*P*_Bon_ = 0.0042).

### Polygenic scoring of apical and septal mass

Finally, we quantified the relationship between cardiovascular outcomes and PRSs for total LVM, apical mass, and septal mass in 424 697 UKBB subjects who did not undergo CMR imaging. The subjects of the original MRI analysis were used to derive a PRS and tested on held-out subjects without MRI imaging. Using a Cox proportional hazards model adjusted by sex, age at acquisition, pulse rate, and hypertension, the PRSs were associated with increased incident cardiomyopathy, acute MI, atrial fibrillation, and ICD implantation. A greater PRS for apical mass conferred higher risk of cardiomyopathy and ICD implantation (*Table 2*). An elevated PRS for total LVM similarly increased risk for these outcomes, while septal mass resulted in increased risk for atrial fibrillation and cardiomyopathy. Subjects at the highest level of apical mass PRS had the greatest risk for cardiomyopathy compared with those at the highest level of total LVM and septal mass PRS.

**Table 2:** Relationship between polygenic risk score and atrial fibrillation, cardiomyopathy, and implantable cardioverter-defibrillator implantation for apical mass, septal mass, and left ventricular mass

Outcome	Hazard ratio [95% confidence interval]		
	PRS (per 1 std)	PRS (90th percentile)	PRS (95th percentile)
Polygenic score for apical mass			
Atrial fibrillation	1.0 [0.99, 1.02] *P* < 0.48	1.02 [0.98, 1.06] *P* < 0.38	1.04 [0.98, 1.10] *P* < 0.16
Cardiomyopathy	1.11 [1.06, 1.15] *P* < 4.4e-7	1.25 [1.11, 1.40] *P* < 4.0e-4	1.44 [1.24, 1.68] *P* < 4.4e-3
ICD implantation	1.08 [1.04, 1.12] *P* < 1.8e-4	1.22 [1.08, 1.37] *P* < 1.0e-3	1.34 [1.15, 1.56] *P* < 2.0e-4
Polygenic score for septal mass			
Atrial fibrillation	1.02 [1.01, 1.04] *P* < 5.8e-3	1.02 [0.98, 1.06] *P* < 0.36	1.07 [0.97, 1.08] *P* < 0.40
Cardiomyopathy	1.08 [1.04, 1.13] *P* < 3.96e-5	1.10 [0.97, 1.24] *P* < 2e-2	1.27 [1.08, 1.49] *P* < 9.5e-4
ICD implantation	1.04 [1.00, 1.08] *P* < 0.12	1.08 [0.95, 1.22] *P* < 0.23	1.14 [0.97, 1.34] *P* < 0.12
Polygenic score for LV mass			
Atrial fibrillation	1.03 [1.02, 1.04] *P* < 4.29e-6	1.05 [1.02, 1.10] *P* < 5.7e-3	1.06 [1.01, 1.13] *P* < 0.01
Cardiomyopathy	1.14 [1.10, 1.19] *P* < 2.46e-11	1.44 [1.29, 1.61] *P* < 1.9e-10	1.37 [1.18, 1.60] *P* < 5.95e-5
ICD implantation	1.09 [1.05, 1.13] *P* < 1.24e-5	1.36 [1.22, 1.52] *P* < 5.21e-8	1.39 [1.20, 1.61] *P* < 1.25e-5

The Cox proportional hazards model is adjusted for sex, age at acquisition, body mass index, pulse rate, and hypertension.

## Discussion

In this study, we leveraged deep learning to identify novel genetic variants of CMR-derived total LVM, apical mass, and septal mass in over 35 000 individuals and established their relationship to incident cardiovascular disease. Notably, apical and septal hypertrophy without increased total LVM both conferred independent excess risk for cardiomyopathy, and apical mass had unique genetic loci compared with prior associations with septal and total LVM. A PRS for apical and septal mass reaffirmed an elevated risk of atrial fibrillation, cardiomyopathy, and ICD implantation. Our findings emphasize the diagnostic importance of focal hypertrophy, which may confer independent as well as additive risk for incident cardiovascular disease. Through downstream *in silico* analysis, we found that genes for apical and septal mass govern cardiovascular structure, function, and implicated in cardiomyopathy, further providing evidence of unique genetic and risk profiles for patterns of hypertrophy.

Whether evaluated categorically as hypertrophy or based on quantitative mass, both increased apical and septal mass resulted in an increased risk for incident cardiomyopathy that was only partially attenuated by global LVH. Subjects with isolated apical cardiomyopathy and isolated septal hypertrophy have a higher risk of cardiomyopathy even without global hypertrophy as defined by current clinical protocols.^35^ The attenuation of the effect of septal mass on incident cardiovascular disease by total LVM parallels the overlapping loci in our genetic association studies for septal mass and LVM. Nevertheless, we identify three genes unique to septal mass, emphasizing that septal hypertrophy may be governed by novel genes in addition to those classically attributed to total LVM in existing studies, such as *TTN* and *MYBPC3.*^10^ Among genes shared between the septal and LVM GWASs, *TTN* is an established gene for familial DCM, *GDF5* promotes cardiomyocyte survival, and loss of *GDF5* is associated with LV dilation and contractile dysfunction.^30^ Of the genes unique to septal mass, *HIVEP3* is a transcription factor that is differentially methylated in HCM;^36^*CEP70* has been implicated in coronary artery disease, MI, and isolated congenital heart disease;^37,38^ and *HMGA1* regulates cardiomyocyte growth with roles in concentric cardiac hypertrophy, MI, and inflammation.^39–41^ Leveraging gene set analysis showed that the genes governing septal mass are involved in morphogenesis of the septum and striated muscle, offering a biological basis for structural cardiac changes. *HIVEP3*, *CEP70*, and *HMGA1* have not been previously associated with LV wall thickness or LVM.^10,15^

Additionally, we uncover variants unique to apical mass, which have not been previously associated with apical HCM^3,32^ or in previous studies of LV wall thickness^15^ and LVM.^14^*MAPT*, which has been previously correlated with LVM^10^ and cross-sectional area of the septal wall,^42^ regulates sarcomere assembly and function;^43^ its dysfunction has been attributed to heart failure with preserved ejection fraction.^44^ Both *CASQ2* and *PLN* are novel variants that were prioritized by eQTL and TWAS and have not been identified in existing GWAS studies of LV phenotypes.^10,12,15^*CASQ2* encodes the protein calsequestrin, with pathologic variants resulting in aberrant calcium release from the sarcoplasmic reticulum, contractile dysfunction, DCM, and catecholaminergic ventricular arrhythmia without structural heart disease.^45,46^*PLN* encodes phospholamban, an integral membrane protein to myocardial calcium pumps, and plays a causal role in dilated, hypertrophic, arrhythmogenic, and familial cardiomyopathies.^47,48^ Notably, *CASQ2* and *PLN* programme proteins that together maintain cardiac calcium homeostasis, which is essential for effective excitation–contraction coupling; gene set analysis further reinforced the significant of *PLN* and *CASQ2* to calcium ion release for cardiomyocyte contraction and electrical coupling. Their dysfunction increases the risk of atrial fibrillation,^49,50^ which is a common and more frequent complication in apical HCM compared with patients with other types of HCM.^3,21^ Additionally, *CASQ2* knockout in mice results in stress-induced arrhythmias and LVH remodelling and contractile dysfunction,^47^ and the lead variant for *PLN* is located in a protein-coding region that is mutated in patients with familial and DCM.^40^ These unique loci suggest that apical hypertrophy may have a genetically distinct pathophysiology, which may contribute to its different clinical outcomes and treatment from other types of HCM. This phenomenon is similarly supported by the elevated risk of cardiovascular outcomes conferred from genetically derived apical mass, suggesting that its genetic drivers are clinically relevant. Our findings highlight the significance of focal hypertrophy to cardiovascular pathophysiology^6–9,51^ and reaffirm the value of characterizing distinct subtypes of LVH.

While smaller clinical cohorts are often underpowered to assess a difference in clinical outcomes, the presence of focal hypertrophy in the UKBB imaging cohort highlights the ability to identify unique subpopulations and their subsequent risk for incident cardiovascular disease. Understanding these patterns of regional hypertrophy may paint a fuller picture of cardiovascular disease outcomes and risk. Limitations of our study include the lack of replication across other external cohorts. Although the UKBB cohort is of mixed-ancestry, European descent predominates, and our results may not be generalizable for individuals of other ancestries.

In conclusion, we analysed the genotypic and clinical significance of apical mass, septal mass, and LVM in over 35 000 UKBB participants. Our results identify 12 loci of clinically observed increased LVM, with 3 loci unique to increased apical mass and an additional 3 loci distinct to septal mass. Our findings suggest that increased apical mass is a significant additional independent risk factor for cardiomyopathy and atrial fibrillation. Such results may be relevant to existing work in characterizing subtypes of HCM, as we find distinct genetic markers and risk profiles for incident cardiovascular disease based on patterns of focal hypertrophy.

## Lead author biography


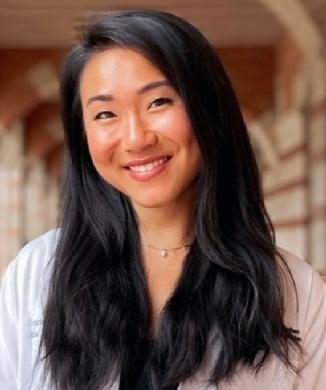


Victoria Yuan is a second-year medical student at the David Geffen School of Medicine at the University of California, Los Angeles. She is excited by research at the intersection of computational methods, cardiovascular imaging, and precision phenotyping. Throughout her first year of medical school, she began this project under Dr David Ouyang at Cedars-Sinai. Prior to medical school, Victoria conducted research as a Fulbright research fellow at the Politecnico di Milano and interned at the Broad Institute of MIT and Harvard. She graduated from Stanford with honours with a BS in Biomedical Computation.

## Supplementary Material

ztae060_Supplementary_Data

## Data Availability

This work analyses UK Biobank data, which are available upon application and approval at https://www.ukbiobank.ac.uk. Phenotypic data will be returned to the UK Biobank.
