# Supplementary material for: Clinical and genetic associations of asymmetric apical and septal left ventricular hypertrophy
Source: Eur Heart J Digit Health. 2024 Aug 9;5(5):591–600. doi: 10.1093/ehjdh/ztae060 (PMC11417484; doi:10.1093/ehjdh/ztae060)
Supplement: ztae060_Supplementary_Data [file ztae060_supplementary_data.docx]

**Supplementary Information**

**Supplementary Figure 1.** Distributions of a) Apical mass versus sex b) Septal mass versus sex c) Baseline diagnoses of aortic valve disease, cardiomyopathy, and hypertension in subjects

**Supplementary Figure 2.** Hazard ratios with 95% confidence intervals for cardiomyopathy, atrial fibrillation, and acute MI. Individual models include the risk conferred by LVM, apical mass, and septal mass when investigated individually as independent variables in models adjusted for sex, age at MRI, BMI, heart rate, and hypertension. Model 2 includes apical mass, LVM, sex, age at MRI, BMI, heart rate, and hypertension, and Model 3 includes septal mass, LVM, sex, age at MRI, BMI, heart rate, and hypertension.

**Supplementary Figure 3.** Quantile-quantile plots of GWAS for a) total LVM b) apical mass c) septal mass

**Supplementary Table 1.** Baseline demographics and diagnoses of the UKBB cohort with CMR imaging and of the entire cohort. Ethnicities are self-reported.

|  | **Cohort with CMR (n=35,623)** | **UKBB Cohort (n=502,490)** |
| --- | --- | --- |
| Age (Mean, [Min, Max]) | 55.0 [40.0,70.0) | 56.5 [37.0, 73.0] |
| Female sex (%) | 18,757 (52.7%) | 27,3450 (54.5%) |
| Male sex (%) | 16,866 (47.3%) | 229,085 (45.6%) |
| BMI (Mean, [Min, Max]) | 26.7 (14.7, 56.6) | 27.4 [12.1, 74.5] |
| White | 34,122 (95.8%) | 472,680 (94.1%) |
| Mixed ethnicity | 167 (0.47%) | 2,958 (0.59%) |
| Asian | 474 (1.3%) | 11,456 (2.3%) |
| Black | 234 (0.66%) | 8,061 (1.6%) |
| Other ethnic group | 167 (0.47%) | 4,558 (0.91%) |
| Ethnicity not available | 87 (0.24%) | 1,878 (0.37%) |
| Heart Rate (Mean, [Min, Max]) | 68.1 [33.0, 174.0) | 69.6 [30.0, 174.0] |
| Hypertension | 2,259 (6.4%) | 15,6045 (31.1%) |
| Cardiomyopathy | 463 (1.3%) | 3,437 (0.68%) |
| Non-rheumatic Aortic Valve Disease | 228 (0.6%) | 7,590 (1.5%) |

**Supplementary Table 2.** Incident outcomes and corresponding field names in UKBB

| **Phenotype** | **Field Name** |
| --- | --- |
| Hypertension | 131286-0.0 |
| Pulse Rate | 102-0.0 |
| Acute MI | 131298-0.0 |
| Atrial Fibrillation | 131350-0.0 |
| Cardiac Arrest | 131346-0.0 |
| Cardiomyopathy | 131338-0.0 |
| ICD Implantation | 41200-0.0, 41260-0.0 |
| Non-rheumatic Aortic Valve Disease | 131325-0.0 |

**Supplementary Table 3.** Genome-wide significant variants with candidate genes, MAF, and function across all phenotypes

| **Phenotype** | **Chr** | **Candidate Gene** | **hg37 Position** | **Ref** | **Alt** | **MAF** | **Function** |
| --- | --- | --- | --- | --- | --- | --- | --- |
| Apex | 1 | **CASQ2** | 116,297,758 | A | C | 0.425 | Intronic |
| Apex | 6 | **PLN** | 118,876,539 | T | C | 0.460 | Exonic |
| Apex | 9 | **RSP19P6** | 83,396,102 | C | T | 0.080 | Intergenic |
| Apex | 17 | MAPT | 43,948,347 | C | T | 0.378 | Intron of non-coding RNA |
| Septal | 1 | **HIVEP3** | 42,007,188 | A | C | 0.4135 | Intronic |
| Septal | 2 | TTN | 179,839,888 | T | C | 0.029 | Exonic |
| Septal | 3 | **CEP70** | 138,172,951 | A | G | 0.490 | Intronic |
| Septal | 4 | **LCORL** | 18,034,463 | G | A | 0.108 | Intergenic |
| Septal | 6 | **HMGA1** | 34,190,104 | A | G | 0.044 | Intergenic |
| Septal | 7 | **HMGN1P19** | 46,620,312 | T | C | 0.447 | Intergenic |
| Septal | 11 | MYBPC3 | 47,366,095 | T | G | 0.139 | Intronic |
| Septal | 20 | **GDF5** | 34,025,756 | G | A | 0.411 | - |
| Total | 2 | TTN | 179,672,414 | T | C | 0.046 | Intronic |
| Total | 2 | **DIRC3** | 218,288,831 | T | A | 0.469 | Intergenic |
| Total | 4 | **LCORL** | 17,917,781 | C | A | 0.300 | Intronic |
| Total | 6 | **BTN3A2** | 26,327,814 | C | T | 0.472 | Intergenic |
| Total | 6 | CDKN1A | 36,646,849 | C | T | 0.317 | Intronic |
| Total | 6 | **AL356534.1** | 127,184,986 | A | G | 0.411 | Intergenic |
| Total | 7 | **HMGN1P19** | 46,620,312 | T | C | 0.447 | Intergenic |
| Total | 10 | **BAG3** | 121,415,685 | A | G | 0.259 | - |
| Total | 11 | **LSP1** | 1,902,768 | A | G | 0.387 | Exonic |
| Total | 11 | MYBPC3 | 47,427,180 | AGA | A | 0.262 | - |
| Total | 12 | **APOLD1** | 12,883,632 | A | AG | 0.257 | Intronic |
| Total | 12 | HMGA2 | 66,343,400 | C | G | 0.457 | Intronic |
| Total | 12 | **SH2B3** | 111,884,608 | C | G | 0.464 | Exonic |
| Total | 13 | TRIM13 | 50,565,104 | A | ACT | 0.025 | Intergenic |
| Total | 16 | FTO | 53,812,770 | A | ATTTT | 0.416 | Intronic |
| Total | 17 | **WNT3** | 44,862,613 | G | A | 0.253 | Intronic |
| Total | 20 | **GDF5** | 34,025,756 | G | A | 0.412 | - |

**Supplementary Table 4.** Results of eQTL analysis using FUMA for lead variants in the GWAS of apical and septal mass

| **Phenotype** | **Chr** | **Candidate Gene** | **hg37 Position** | **Ref** | **Alt** | **Tissue** | **p-value** |
| --- | --- | --- | --- | --- | --- | --- | --- |
| Apex | 1 | CASQ2 | 116,297,758 | A | C | Coronary Artery | 8.51e-10 |
| Apex | 1 | CASQ2 | 116,297,758 | A | C | Atrial Appendage | 3.95e-8 |
| Apex | 1 | CASQ2 | 116,297,758 | A | C | Left Ventricle | 1.21e-9 |
| Apex | 6 | PLN | 118,876539 | T | C | Aorta | 2.84e-8 |
| Apex | 6 | PLN | 118,876539 | T | C | Atrial Appendage | 1.5e-4 |
| Apex | 17 | MAPT | 43,948,347 | C | T | Left Ventricle | 7.80e-5 |
| Septal | 3 | CEP70 | 138,172,951 | A | G | Left Ventricle | 5.38e-7 |
| Septal | 3 | CEP70 | 138,172,951 | A | G | Coronary Artery | 1.0e-5 |
